# Lymphocytes and Trogocytosis-Mediated Signaling

**DOI:** 10.3390/cells10061478

**Published:** 2021-06-12

**Authors:** Jim Reed, Madison Reichelt, Scott A. Wetzel

**Affiliations:** 1 Division of Biological Sciences, University of Montana, Missoula, MT 59812, USA; Jim.Reed@seattlechildrens.org (J.R.); madison.reichelt@umconnect.umt.edu (M.R.); 2 Center for Environmental Health Sciences, University of Montana, Missoula, MT 59812, USA

**Keywords:** trogocytosis, intracellular signaling, intercellular transfer, CD4^+^ T cells, natural killer cells, auto-presentation, cytokine, differentiation, immune response, immune synapse

## Abstract

Trogocytosis is the intercellular transfer of membrane and membrane-associated molecules. This underappreciated process has been described in a variety of biological settings including neuronal remodeling, fertilization, viral and bacterial spread, and cancer, but has been most widely studied in cells of the immune system. Trogocytosis is performed by multiple immune cell types, including basophils, macrophages, dendritic cells, neutrophils, natural killer cells, B cells, γδ T cells, and CD4^+^ and CD8^+^ αβ T cells. Although not expressed endogenously, the presence of trogocytosed molecules on cells has the potential to significantly impact an immune response and the biology of the individual trogocytosis-positive cell. Many studies have focused on the ability of the trogocytosis-positive cells to interact with other immune cells and modulate the function of responders. Less understood and arguably equally important is the impact of these molecules on the individual trogocytosis-positive cell. Molecules that have been reported to be trogocytosed by cells include cognate ligands for receptors on the individual cell, such as activating NK cell ligands and MHC:peptide. These trogocytosed molecules have been shown to interact with receptors on the trogocytosis-positive cell and mediate intracellular signaling. In this review, we discuss the impact of this trogocytosis-mediated signaling on the biology of the individual trogocytosis-positive cell by focusing on natural killer cells and CD4^+^ T lymphocytes.

## 1. Trogocytosis

Trogocytosis the direct, intercellular transfer of membrane and membrane-associated molecules in a contact-dependent manner. This term was originally coined by Brown and colleagues to describe the cytopathogenicity of the parasitic amoeba *Naegleria fowleri* [1], and it was later employed by Joly and Hudrisier to describe a comprehensive model of intercellular transfer of membrane and membrane molecules between immune cells [2].

Most studies on trogocytosis have focused on interactions of immune cells. Trogocytosis has been observed in CD4^+^ [3,4,5,6,7,8,9,10,11], CD8^+^ [9,12,13,14,15], and γδ [16] T cells, B cells [17,18,19], Natural Killer (NK) cells [8,20,21,22], basophils [23], macrophages [24,25], neutrophils [26,27,28], and dendritic cells [29,30]. However, trogocytosis is a more general cellular phenomenon that is not limited to immune cells [31]. It has been described in a variety of cellular interactions including microglial presynaptic remodeling [32,33], trogocytosis of oocyte proteins by sperm before fertilization [34], during embryonic development [35,36,37], stromal cell protein trogocytosis by cancer cells [38], erythrocyte interactions with epithelia [39], and parasite interactions with neutrophils to help evade immune detection [40,41,42,43]. It has also been implicated in cell–cell spread of intracellular bacteria such as *Francisella tularensis* [44] and viral pathogens [10,45,46].

The initial descriptions of trogocytosis date back to the early 1970s [47,48,49,50]. In 1972, Cone et al. detected allogeneic MHC class II molecules on adoptively transferred T cells in mice [47]. It was postulated at the time that T cells had picked up these allogeneic determinants in vivo, that the MHC molecules were integrated into the T cell surface, and that they were similar in composition to the membrane-bound form found on B cells [48,49,50]. That conclusion, as to the source of the MHC class II on the T cells, was supported by subsequent work showing that murine T cells do not endogenously express MHC class II molecules [51]. In 1973, Bona et al. published the first report of antigen (Ag) transfer from antigen-presenting cells (APCs) to lymphocytes. In that paper, they found that B lymphocytes acquire LPS from macrophages in a B cell receptor-dependent manner and that this required direct cellular contact between the B cell and LPS-loaded macrophage [52]. These initial papers were followed several years later by the work of Hudson and Sprent, who reported that, after adoptive transfer of activated T cells into irradiated allogeneic hosts, the transferred T cells had detectable levels of B-cell-derived IgM on their surface [50]. Subsequent reports described the transfer of MHC:peptide to T cells from APC both in vitro [53,54,55] and in vivo [56].

For both T cells [3,6,15,45,57,58,59] and natural killer cells [60,61,62,63,64,65], trogocytosis occurs at the immunological synapse. This results in the trogocytosis of cognate ligands and additional molecules enriched within the synapse including CD80 [6,66,67], CD86 [45,68,69,70], OX40-L [7], PDL-1 [14], and adhesion molecules such as ICAM-1 [71] onto T cells. The transfer of membrane lipids [72,73] strongly suggests that trogocytosis includes the intercellular transfer of membrane fragments. T cells also capture nonspecific MHC, e.g., CD4^+^ T cells capture MHC class I molecules [8,54,74] or irrelevant MHC molecules [75]. This has potential implications for immune modulation, as discussed below.

With our increased understanding of trogocytosis, it has become a tool in both basic research and the clinic. The detection of trogocytosis by flow cytometry has proven to be a useful tool in the discovery of antigen and detection of antigen-specific lymphocytes. It has been used to identify antigen-specific B and T cells [76,77] and virus-specific T cells when antigenic epitopes are not known [78]. Recently, it was also employed by Li et al. to detect and subsequently identify cognate antigenic epitopes for specific, disease-associated T cell antigen receptors (TCRs) [58]. Trogocytosis has also been used to engineer surface expression on NK cells of molecules not endogenously expressed by the cells [79]. As they are further developed, these tools hold the potential to become clinically important in infectious disease, cancer, and autoimmune disease detection and treatment.

## 2. Mechanism of Trogocytosis

Defining the mechanism of trogocytosis has proven somewhat elusive. The efficiency of trogocytosis is impacted by a variety of factors. It is an active process, requiring both antigen recognition and cognate receptor signaling [6,18,60]. In T cells, trogocytosis is Ag-specific [3,4,14,58,80], and blocking proximal TCR signaling through ZAP-70 significantly reduces the rate of trogocytosis [12]. Similarly, treatment of cells with the MEK/ERK inhibitor U0126 or the Src family kinase inhibitor PP2 significantly inhibits trogocytosis [18,81]. Varying the strength of TCR signaling by modulating Ag dose positively correlates with the extent of trogocytosis [3,12,82]. Our unpublished results also show that trogocytosis by CD4^+^ T cells is dependent on the affinity of the TCR for MHC:peptide, as APC loaded with lower-affinity peptide analogues leads to a significant reduction in trogocytosis (Reichelt and Mengistu, manuscript in preparation). We have also reported that, while antibody blockade of CD80 had minimal impact on trogocytosis of MHC class II:peptide, blocking of MHC class II or CD3 significantly reduced trogocytosis of MHC class II:peptide [6].

The activation state of the cell impacts trogocytosis, as activated T cells are much more efficient at performing trogocytosis than naïve T cells [66,68,73,83,84,85,86]. This may be due to the increased size of activated cells, as they have a larger region of membrane contact with APC, or due to increased avidity of the activated cell, as naïve and activated T cells have identical antigen receptors, but have increased expression of adhesion and costimulatory molecules, such as CD28, LFA-1, and CD44, which stabilizes the immune synapse and may facilitate trogocytosis [68,87].

Trogocytosis is contact-dependent and requires actin cytoskeletal rearrangement [60]. Treatment of cells with the actin polymerization inhibitors latrunculin or cytochalasin D severely limits trogocytosis [9,87]. When cells APC and lymphocytes are co-cultured, trogocytosis occurs efficiently, but it is significantly reduced or completely absent when cells are separated in transwell dishes [14,88,89]. These findings are corroborated by transmission electron microscopy (TEM) studies that have shown that, during trogocytosis, small regions (~50–95 nm in diameter) of the opposing plasma membranes may fuse [65,72]. Interestingly, early studies found that, while trogocytosis involved membrane and membrane-associated proteins [72,73,90], it did not include transfer of cytoplasmic contents between cells [72]. However, inner leaflet associated proteins, such as H-Ras, are transferred during trogocytosis [91]. The transfer of H-Ras, in both T cells and NK cells, initiates intracellular signaling leading to elevated phosphorylated ERK 1/2 (pERK), increased IFNγ, TNFα, and proliferation, and enhanced NK killing [91]. This signaling is distinct from trogocytosis-mediated signaling discussed below.

Trogocytosis is mechanistically distinct from interactions with APC-derived exosomes as it involves the transfer of large (up to several μm^2^) membrane fragments, and blocking exosome formation in DC with the ATPase inhibitor CMA only slightly decreases the amount of trogocytosis [14]. Similarly, incubation with APC-derived cellular debris formed by repeated freeze–thaw cycles does not result in efficient capture of membrane proteins by CD4^+^ T cells [6].

As with T cells, NK cell trogocytosis, from both activating and inhibitory immune synapses [64], is largely mediated by receptor–ligand interactions. For example, Ly49A mediates the trogocytosis of D^b^ [65], and NKG2D mediates the trogocytosis of MICA [92], MICB [62], viral ligands [22], and Rae-1δ [81] from target cells.

## 3. Current Model of Trogocytosis

**Figure 1:**
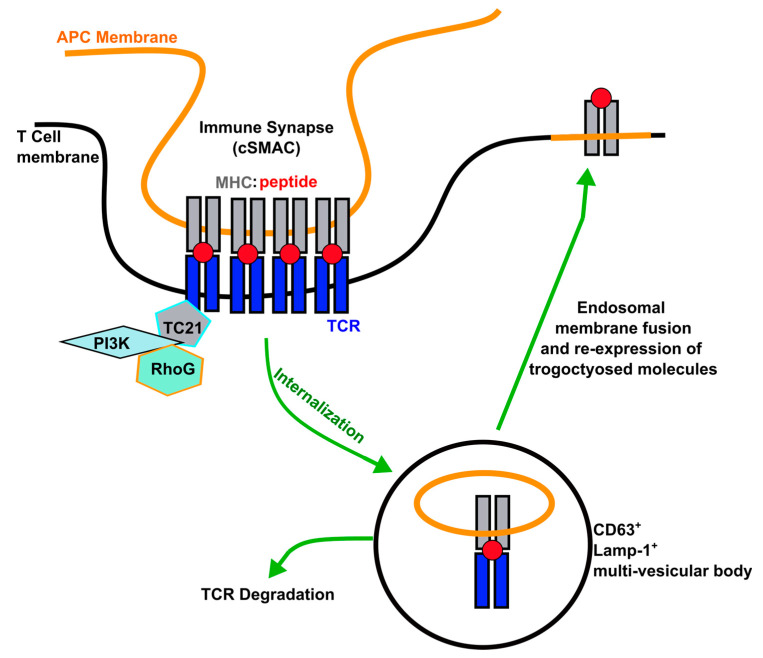
Model of trogocytosis event. T cells phagocytose APC membrane and membrane molecules from central Supramolecular Complex (cSMAC) of the immune synapse in a TC-21- and RhoG-dependent mechanism. The trogocytosed molecules traffic through multivesicular bodies where the TCR is sorted for degradation, and the endosome membrane fuses with phagocytosed APC membrane. The trogocytosed molecules traffic the plasma membrane for re-expression. Modified from [75,94].

The current model of trogocytosis proposes that trogocytosis by CD4^+^ T cells (and by extension CD8^+^ T cells and NK cells) is an RRas2- and RhoG-dependent phagocytosis event (Figure 1) [93,94,95]. The APC-derived membrane and membrane proteins are transferred via phagocytosis at the immunological synapse during TCR downmodulation [94]. Consistent with this model, trogocytosis by RhoG^−/−^ CD4^+^ T cells is significantly reduced [75]. The trogocytosed APC membrane fragments are internalized into endosomes, as pre-treatment of T cells with the vacuolar ATPase CMA to inhibit endosomal formation and trafficking significant decreases trogocytosis [14]. In T cells, it has been shown that the internalized MHC class II partly colocalizes with Lamp-1^+^ and CD63^+^ vesicles, suggesting that the trogocytosed molecules traffic through multivesicular bodies on the way to integration into the plasma membrane [75]. Once internalized by the T cell, recycling endosomes containing acquired APC fragments fuse with the T cell plasma membrane, resulting in APC-derived molecules being displayed on the T cell surface in their native topological orientation [94,95].

This phagocytosis/endocytosis–surface re-expression model is consistent with the results of Sprent and colleagues who found trogocytosed GFP-tagged MHC class I L^d^ molecules are internalized by CD8^+^ T cells before surface re-expression [82]. It is also supported by studies showing that the broad endocytosis inhibitor methyl-β-cyclodextrin inhibits NKG2D-mediated, Rae-1 trogocytosis by NK cells [81]. Chlorpromazine (an inhibitor of clathrin-dependent endocytosis), but not filipin (an inhibitor of caveolae-dependent endocytosis) also inhibits NK cell trogocytosis of Rae-1 [81]. These results are consistent with the Martinez–Martin model and suggest that, at least in NK cells, receptor-mediated trogocytosis is coupled with clathrin-dependent, receptor downmodulation [81]. The molecular events that mediate the transfer of trogocytosed membrane proteins from the endocytosed APC membrane to the recycling endosomal membrane, which results in plasma membrane integration of trogocytosed proteins in the correct topological orientation, remain to be elucidated.

## 4. Trogocytosis-Positive Cells as Antigen Presenting Cells

The presence of ectopically expressed membrane proteins on the trogocytosis-positive cell imparts new biological properties to the trogocytosis-positive recipient of these molecules. One of the best studied examples of this is the ability of a trogocytosis-positive T cell to act as an antigen-presenting cell. Studies with rat T cells, which endogenously express MHC class II molecules, found that, when a peptide antigen was added to these cells, it resulted in anergy induction due to T–T antigen presentation [96,97]. This was likely due to a lack of costimulatory molecules expressed by the presenting T cell. However, because trogocytosis involves the capture of APC membrane proteins from the immunological synapse, including costimulatory and adhesion molecules, there has developed a significant body of literature related to trogocytosis-positive T cells acting as APCs themselves. Antigen presentation by trogocytosis-positive cells has been shown to modulate the phenotype and response of responding cells.

The response initiated by trogocytosis-positive T cells presenting antigens is largely a function of the activation state of the responding T cell. Antigen presentation by trogocytosis-positive cells activates responding naïve T cells [10,88,98,99], but has been reported to induce cell death in previously activated responders [100]. For example, Helft et al. showed in vivo that, when the responding T cell was naïve, trogocytosis-mediated T–T antigen presentation lead to activation and proliferation of the responding cell, while previously activated responders underwent apoptosis [100]. Several reports have shown that, after trogocytosis of MHC class I, trogocytosis-positive T cells (both CD4^+^ and CD8^+^) may induce cytokine production from responders [101]. TEM images demonstrate that an immune synapse forms between trogocytosis-positive cytotoxic T lymphocytes (CTL) and responding CTL [72] and supports the findings that the trogocytosis-positive cells can be killed by CTL (fratricide) [82,84,101,102,103].

Attenuation of the immune response via antigen presentation by trogocytosis-positive T cells has been observed using T cells from multiple myeloma patients. In vitro incubation of patient cells with myeloma cell lines led to trogocytosis of CD86 and the immunosuppressive molecule HLA-G [70]. HLA-G^+^ trogocytosis-positive cells are functionally comparable to thymic-derived natural T regulatory cells (nT_reg_), inhibiting proliferation of responding T cells [104,105]. In samples directly from patients, a significantly higher frequency of circulating T cells had detectable HLA-G and CD86 compared to healthy controls, and the increase in the frequency of these trogocytosis-positive cells was associated with poor prognosis [70]. In another study using human T cells, Game et al. showed that, in an allogenic response, trogocytosis-positive cells can stimulate both naïve allogenic and autologous T cells to proliferate [88].

Presentation by trogocytosis-positive effector CD4^+^ T cells vs. trogocytosis-positive T_reg_ results in differential regulation of CD4-mediated immune responses. A comparison of CD4^+^ T cell effector subsets is outlined in Table 1. In general, antigen presentation by trogocytosis-positive T_reg_ attenuates responses and presentation by trogocytosis-positive effector CD4^+^ T cells potentiates them [98]. In addition to trogocytosis conferring regulatory functions to non-T_reg_ cells [14,105,106,107,108,109,110], T_reg_ cells themselves display high rates of Ag-specific trogocytosis [57]. CD80 and CD86 have been detected on the surface of both thymic-derived natural regulatory T cells (nT_reg_) and antigen-induced, peripheral induced regulatory T cells (iT_reg_) [106], and it has been proposed that trogocytosis-positive T_reg_ cells use trogocytosed molecules to form connections with activated T cells, thus enhancing their suppressive activity [109,110]. Consistent with this, trogocytosis-positive T_reg_ cells or T_H_ cells displaying trogocytosed immune-suppression associated molecules, such as HLA-G, show enhanced suppressive capabilities [98,107,110,111,112,113]. Ectopic expression of HLA-G via trogocytosis may be important clinically in cancer as it may lead to wider immunosuppression and tumor growth [114].

Antigen presentation by trogocytosis-positive T cells does not universally lead to attenuation of an immune response. Xiang and colleagues have shown that CD4^+^ T cells capture bystander MHC class I molecules from APCs, and they can induce CD8^+^ T cell activation [8,11,74,109]. They have hypothesized that class I trogocytosis by CD4^+^ T cells and subsequent presentation to CD8^+^ T cells may play an important role in CD4^+^ T cell “licensing” of CTL activity [74,115] and help solve the complex kinetic conundrum whereby CD4^+^ T cells, dendritic cells, and CD8^+^T cells must interact sequentially or simultaneously to allow CD4^+^ licensing of CD8^+^ T cells. More recently, Boccasavia et al. found that trogocytosis-positive and OT-II TCR transgenic T cells can present antigen and stimulate naïve T cells in an antigen-specific manner [75]. When the phenotype of the cells was examined, the responding T cells secreted IL-2 and TNFα, and they upregulated CCR6, a chemokine receptor associated with a T_H_17 phenotype. Interestingly, the trogocytosis-positive stimulating T cells downregulated CCR6 and upregulated Foxp3. After a 6-day coculture, the frequency of Foxp3^+^ cells among the trogocytosis-positive antigen-presenting T cells was almost sixfold higher than the responders, and the frequency of RORγt^+^ IL-17A^+^ cells in the responder population was sevenfold higher than in the trogocytosis-positive presenting population. This polarization of the trogocytosis-positive population to T_reg_ cells and responders to T_H_17 was also observed in vivo [75]. The number of Foxp3^+^CD25^+^ CD4 T_reg_ cells was directly proportional to the number of antigen-presenting cells in the initial T-APC culture, while the number of T_H_17 cells was indirectly proportional to number of APC. The authors concluded that an abundance of antigen-bearing APC favors the differentiation of T_reg_, while limiting number of APC favors T_H_17 differentiation because that favors T–T interactions and presentation by trogocytosis-positive T cells [75].

As with T cells, the presence of trogocytosed molecules on NK cells can mediate NK–NK cell interactions, leading to alterations in function and cytolytic activity of both the trogocytosis-positive NK cell and the responding cell. For example, NKG2D^+^ NK cells capture the activating ligand MICA from target cells, resulting in the subsequent activation of bystander NK cells and triggering of cytolytic granule release those cells. This ultimately leads to the death of the MICA^+^ NK cells via fratricide [92]. Similarly, NKG2D^+^ NK cells can trogocytose the activating ligand Rae-1δ from target cells, and the Rae-1δ^+^ NK cells themselves become the target of NK cells killing, both in vitro and in vivo [81]. This has been proposed as a mechanism of controlling the frequency of activated NK cells. Unexpectedly, NK1.1^+^ NK cells trogocytose MHC class II:peptide complexes from dendritic cells, and these trogocytosis-positive NK cells can subsequently negatively regulate CD4^+^ T cell activity both in vitro and in vivo [21].

Thus, both trogocytosis-positive T cells and trogocytosis-positive NK cells can present antigens to bystander cells and modulate immune responses [8,10,74,75,81,92,98,100,105,108,116,117,118,119]. Collectively, the findings outlined above underscore the fact that trogocytosis and the subsequent presentation of acquired molecules are biologically significant events and play an underappreciated role in the regulation of immune responses. The consequences of such presentation correlate with the nature of the acquired molecules and phenotype of the trogocytosis-positive and responding cell.

## 5. Membrane Topology of Trogocytosed Molecules

Trogocytosed molecules are found in a focused spot on the CD4^+^ T cell surface [3,6,98], and the ability of trogocytosis-positive T cells to present antigens, in the context of other acquired molecules, to responding T cells confirms that they remain fully functional [8,10,11,74,98,99,105,112,117,120,121]. One conclusion based upon antigen presentation by trogocytosis-positive T cells or NK cells is that the molecules transferred from APCs must be accessible on the surface of the cell and in the correct topological orientation to interact with receptors on the responder cells. As we have outlined previously [4] and as covered by another review on this issue [122], trogocytosed molecules may be integrated into the trogocytosis-positive cell membrane or could be cell-associated via tethered, extracellular membrane vesicles. The question of integration vs. vesicle tethering is not mutually exclusive, as a study from Hudrisier and colleagues found that, while 76% of trogocytosed Fc gamma Receptor (FcγR) on T cells was eluted from the cell by mild acid treatment, approximately 20% of the detected FcγR was not, and they concluded that this was consistent with membrane integration of the trogocytosed FcγR [123].

Several lines of evidence support integration into the trogocytosis-positive cell membrane including flow cytometry and light microscopy studies, which have shown that extracellular epitopes are accessible on intact cells and intracellular epitopes are inaccessible unless the cells are detergent-permeabilized [3,6,65]. Similarly, a mouse cytomegalovirus (MCMV) protein, m157, trogocytosed from infected cells is integrated into an NK cell membrane via a glycosylphosphatidylinositol (GPI) linker [22]. Early transmission electron microscopy data showed that trogocytosed H-2Dd was found integrated into the membrane of trogocytosis-positive CTL [72]. Recently published super-resolution light microscopy and transmission electron microscopy data from Boccasavia et al. have very clearly established that trogocytosed MHC:peptide molecules on T cells are associated with the plasma membrane and are in the native topology [75].

## 6. Trogocytosis-Mediated Signaling

While the ability of trogocytosis-positive T cells to present trogocytosed molecules to other T cells is well documented, much less is known about the biological consequences of trogocytosis on the individual trogocytosis-positive cell. Membrane proteins acquired from APCs are detectable for several days after removal of the APC on NK cells [65] and CD4^+^ T cells [3,5,6,59,75]. The presence of trogocytosed molecules has the potential to significantly impact the biological functions of cells. For example, human NK cells express the chemokine receptor CCR7 on their surface as a function of trogocytosis [79,124], as they do not endogenously express this protein [79]. The trogocytosed CCR7 is able to bind the ligand and signal the NK cell, mediating chemotaxis both in vitro and in vivo. In transwell experiments, there was approximately a sixfold increase in the migration of these CCR7^+^ NK cells toward the CCR7 ligands CCL19 and CCL21 [79,124]. After adoptive transfer into nude mice, these CCR7^+^ NK cells migrated to lymph nodes at a significantly higher rate than the CCR7^–^ NK cells [79]. Thus, the trogocytosis of CCR7 led to new functionality of NK cells. Another example of the potential biological impact of trogocytosis is the transfer of CCR5 from peripheral blood mononuclear cells to endothelial cells during transendothelial migration. It is hypothesized that this may allow the endothelial cells to be infected by a macrophage-tropic strain of HIV-1 [125]. Hudrisier et al. also reported that FcγR is trogocytosed by CD4^+^ T cells and it efficiently binds to immune complexes, although it does not induce detectable intracellular signaling [123].

One underappreciated possibility is that trogocytosed molecules interact with cognate receptors on/in the trogocytosis-positive cell and mediate intracellular signaling, so-called *trogocytosis-mediated signaling*. Trogocytosis-mediated signaling has been described in nonimmune systems. In a study using transfected CHO and HeLa cells expressing fluorescently tagged CD30 or CD30L, Nakashima et al. showed that CD30^+^ cells trogocytose CD30L from neighboring cells in an actin-dependent, clathrin-independent manner. This was associated with CD30 internalization, and the CD30–CD30L complexes traffic to the lysosome where they colocalize with TRAF molecules, consistent with active, trogocytosis-mediated signaling occurring within in the trogocytosis-positive cell. Ca^2+^ flux data helped confirm that, indeed, trogocytosis-mediated CD30 signaling is driven by trogocytosed CD30L in trogocytosis-positive cells [126].

**Figure 2:**
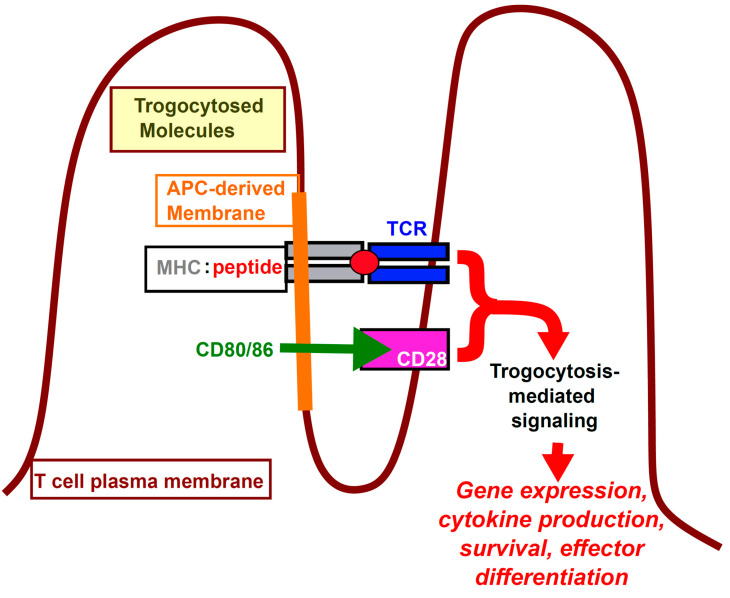
Simplified schematic of CD4^+^ T cell trogocytosis-mediated signaling. T cell membrane projections with APC-derived trogocytosed molecules (left) engage cognate receptors on the surface of the T cell (right) and induce TCR + co-stimulation signaling.

**Figure 3:**
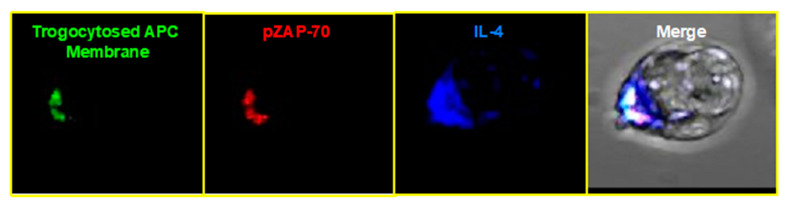
IL-4 is polarized toward trogocytosed MHC:peptide and active TCR signaling (phosphorylated ZAP-70). TCR transgenic cells were incubated for 90 min with APCs expressing GFP-tagged MHC:peptide. Cells were recovered, permeabilized, and stained for phosphorylated ZAP-70 and IL-4.

Trogocytosed class II MHC:peptide molecules are retained in punctate areas on the CD4^+^ T cell surface of [3,6,98] for 5–6 days after APCs are removed [3,4,6,59,75], and they colocalize with the TCR [3,6,59]. Similarly, MICB trogocytosed by NK cells colocalizes with its cognate receptor, NKG2D [127]. Strikingly, on CD4^+^ T cells, there is specific accumulation of active signaling molecules, such as phosphorylated ZAP-70 (pZAP-70), phosphorylated Lck (pLck), and phosphorylated ERK 1/2 (pERK), at the site of trogocytosed MHC:peptide and accumulated TCR [3,6,59], strongly suggesting that the MHC:peptide is engaging the TCR and inducing signaling. (Figure 2) The actin cytoskeleton is polarized toward the engaged TCR and arranged in a ring reminiscent of the peripheral supramolecular activation complex (pSMAC) [6] of the immunological synapse [128,129]. Effector cytokines, such as IL-4, are also specifically polarized towards the trogocytosed trogocytosed MHC:peptide engaged TCR (Figure 3) [59]. No such accumulation is observed in trogocytosis-negative cells. These results are consistent with the trogocytosed, APC-derived molecules on the T cell directly mediating intracellular signaling by interacting with specific receptor(s) on the T cell surface of the trogocytosis-positive cell.

Trogocytosis-mediated signaling has also been reported to occur with NK cells. On NK cells, the activating ligand MICB colocalizes with NKG2D and markedly reduces the cytolytic activity of these cells [127]. This is consistent with a report that chronic MICA exposure impairs NK and CTL cytolytic ability [130]. Interestingly, NKG2D-dependent cytolytic activity was markedly reduced on the MICB^+^ NK, although perforin levels were unchanged and killing via LY49D was unaffected [127]. Similarly, Ly49H on NK cells specifically binds to the MCMV protein m157. NK cells trogocytose m157, which is attached to the NK plasma membrane via a GPI linker. The m157^+^ NK cells are hyporesponsive in vitro and in vivo, and it is proposed that the trogocytosed m157 engages LY49H and sustains signaling through LY49H, thus rendering the cell hyporesponsive [22].

After trogocytosis, trogocytosis-positive CD4^+^ T cells preferentially survive over a 5-day culture in vitro after removal of APCs [3]. Immediately after the T cells are removed from APCs, the trogocytosis-negative cells represent almost two-thirds of the CD4^+^ cells. Over several days, the frequencies of trogocytosis-positive and trogocytosis-negative cells reverse such that trogocytosis-positive cells make up more than 82% of the viable CD4^+^ T cells and the trogocytosis-negative cells represent less than 18% of viable CD4^+^ cells on day 3. The level of trogocytosed MHC:peptide associated with the cell remains fairly constant on the trogocytosis-positive cells over 5 days, and there is minimal CFSE (5-(and 6)-carboxyfluorescein diacetate succinimidyl ester) dilution (whose intensity is halved with each cell division), suggesting that, under those conditions, the trogocytosis-positive cells are selectively surviving, but not dividing [3]. These results strongly support the hypothesis that sustained, trogocytosis-mediated signaling plays a central part in the selective survival of the trogocytosis-positive cells.

Consistent with sustained, trogocytosis-mediated signaling, Zhou et al. showed that sustained, trogocytosis-mediated signaling alters biological outcomes in trogocytosis-positive cells. In that study, trogocytosis-positive CD4^+^ T cells had elevated NF-κB (p50/p50) and AP-1 activity 24 h after APC removal [131]. These cells had elevated phosphorylated STAT-5 1 h after APC removal, but the levels were back to background by 3 h. Interestingly, the trogocytosis-positive cells also developed a unique cytokine profile expressing IL-2, IL-9, and IFNγ after removal of the APC [131], suggesting that trogocytosis-mediated signaling induces qualitatively different signaling compared to signaling induced by interactions with the APC at the immunological synapse.

**Table 1:** CD4^+^ T cell effector subsets.

Effector Subset	Transcriptional Regulator	Characteristic Cytokine	Immunological Functions
T_H_1 [132,133]	T-bet [134]	IFNγ	Intracellular pathogens and cancer
T_H_2 [132,133]	Gata-3 [135]	IL-4	Helminthes and parasites
T_H_17 [136,137]	ROR_γ_t [138]	IL-17	Extracellular pathogens, neutrophil activation, inflammation
T_FH_ [139,140,141]	Bcl6 [142]	IL-21	Mediate germinal center reaction (e.g., somatic hypermutation)
T_H_22 [143]	AhR [144,145]	IL-22	Extracellular bacteria, skin protection
T_Reg_ [146,147]	Foxp3 [148,149]	TGF-β	Regulate immune responses

When the potential impact of trogocytosis-mediated signaling on effector cytokine production was examined, it was observed that 5 h after removal of APCs, trogocytosis-positive cells expressed significantly lower levels of IFNγ and significantly higher levels of IL-4 compared to trogocytosis-negative cells in the same culture [59]. After removal of APCs, the frequency of trogocytosis-positive IL-4^+^ cells increased sevenfold between 5 h and 72 h, reaching 70% of the population [59]. Robust GATA-3 expression was detected in trogocytosis-positive cells by 72 h, but a similar T_H_2 phenotype was not observed with anti-CD3 + anti-CD28 stimulation. Taken together, these results are in agreement with the earlier work showing that trogocytosis-mediated signaling is qualitatively different [131], participates in cell effector subset differentiation, and is not simply boosting intracellular signaling globally. While the frequency of trogocytosis-positive IL-4^+^ cells increased, the frequency of trogocytosis-positive IFNγ-producing cells decreased from 10% at 5 h to 0.5% at 72 h after APC removal. To confirm that signaling was being driven by trogocytosed molecules and not residually from the T–APC interaction, cells were treated with the reversible Src family kinase inhibitor PP2 to halt TCR signaling. When the PP2 was subsequently washed out, signaling resumed in the trogocytosis-positive, but not trogocytosis-negative cells, consistent with signaling being induced by trogocytosed molecules, i.e., trogocytosis-mediated signaling. The frequency of trogocytosis-positive IL-4^+^ cells from PP2-treated cultures rebounded to levels equal to untreated cells by 24 h after PP2 removal. There was robust IL-4 production and GATA-3 expression observed in trogocytosis-positive, but not trogocytosis-negative, cells 72 h after PP2 treatment. By comparison, the expression of IFNγ did not resume in either trogocytosis-positive or trogocytosis-negative cells. Somewhat unexpectedly, in vitro polarized trogocytosis-positive T_H_1 cells began expressing IL-4 and GATA-3, while simultaneously decreasing expression of IFNγ and T-bet [59], further supporting the conclusion that trogocytosis-mediated signaling promotes T_H_2 differentiation. Differentiation of trogocytosis-positive T cells to a T_H_2 phenotype was also observed in vivo during an immune response.

The increased frequency of T_H_2 cells after APC removal partially reflects differences in trogocytosis efficiency as in vitro polarized T_H_2 cells are more efficient than in vitro polarized T_H_1 or nonpolarized cells at performing trogocytosis [59]. The differences in trogocytosis efficiency between polarized T_H_1 and T_H_2 cells may reflect morphological differences in their immunological synapses. At low Ag concentrations, T_H_1 synapses form the classical “bull’s eye” shape, while T_H_2 cells form multifocal synapses [150]. In separate live-cell imaging experiments, it has been observed that small “packets” of MHC:peptide are transferred from APCs to nonpolarized T cells from the immunological synapse, which are subsequently found in punctate spots at the distal pole of the T cell [151], as discussed below. It is inviting to speculate that the multifocal synapses formed by T_H_2 cells facilitate trogocytosis more efficiently than the synapses formed by T_H_1 cells, although this has not been formally tested.

The concept of auto-presentation is not new as, three decades ago, it was reported that T cell self-presentation of cognate peptide induces killing of the individual cell [152,153]. Auto-presentation of trogocytosed molecules may play a role in signal “summing” [154], i.e., repeated short-duration encounters resulting in T cell activation [155,156]. The strength, duration, and “summation” of signaling significantly impacts T helper differentiation [157,158,159,160]. It has been shown that weaker TCR signaling drives early T cell IL-4 production [159,161,162,163]. In contrast, IFNγ production and T_H_1 differentiation have been shown to require strong TCR-signaling [164,165,166]. Since only a fraction of the APC molecules localized to the immunological synapse are transferred to the T cell, trogocytosis-mediated signaling is presumably weaker than synaptic signaling. Thus trogocytosis-mediated signaling could further promote IL-4 production, consistent with the increased levels of IL-4 observed in trogocytosis-positive cells over a 72-h incubation. The observed T_H_1 to T_H_2 conversion further supports this model, as weak TCR signaling drives T_H_2 differentiation, even under T_H_1-polarizing conditions [167].

## 7. Potential Role of Trogocytosis-Mediated Signaling in CD4^+^ Cell Differentiation: T_FH_

**Figure 4:**
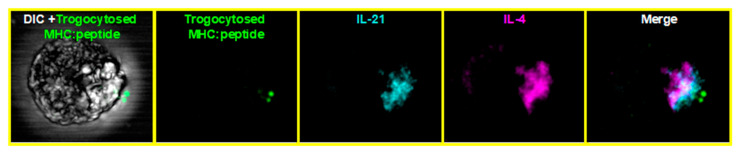
IL-4 and IL-21 were polarized toward trogocytosed MHC:peptide TCR transgenic cells incubated for 90 min with APCs expressing GFP-tagged MHC:peptide. Cells were recovered, permeabilized, and stained for intracellular IL-21 and IL-4.

Beyond driving a T_H_2 phenotype, continual trogocytosis-mediated signaling may aid in the generation of follicular helper T cells (T_FH_) or CD4^+^ memory cells, since sustained TCR signaling through repeated antigen encounter is required for their differentiation [157,168]. The conversion of T_H_2 to a T_FH_ phenotype takes between 5 and 7 days in vivo [169]; thus, examination of the phenotype of trogocytosis-positive cells at extended time-points is likely necessary to address this possibility. Our preliminary, unpublished data have shown that, after 5 days, the trogocytosis-positive cells begin expressing Bcl6, CXCR5, and IL-21, all of which are characteristics of T_FH_ differentiation. IL-21 appears to be polarized toward the trogocytosed molecules in a pattern similar to IL-4 (Figure 4). Studies are currently underway in our lab to examine the potential role of trogocytosis-mediated signaling in the generation of T_FH_ cells.

## 8. Potential Role of Trogocytosis-Mediated Signaling in CD4^+^ Memory Cell Differentiation

Interestingly, as mentioned above, trogocytosed molecules move from the T–APC interface to a region of the T cell directly opposite the immune synapse, i.e., the distal pole complex [170,171], more than 80% of the time. These trogocytosed patches are arranged in a circular pattern at the distal pole, forming a “trogocytosis crown”. The reason for this segregation of the trogocytosed molecules to the distal pole is unknown, but this observation raises the intriguing possibility that the trogocytosed molecules may play a role in asymmetric T cell division. In asymmetric division, proteins and signaling molecules are unevenly segregated in a bipolar manner that reflects differential cell fate (effector versus memory) in daughter cells [172,173]. Early models of asymmetric division have suggested that signaling of different strength or qualitatively different signaling from the immune synapse and the distal pole complex may mediate CD8^+^ T cell asymmetric division and cell fate (effector vs. memory). The integration of differential signaling from the immune synapse and from trogocytosed molecules may help establish asymmetric T cell division and subsequent differential T cell fate, as suggested by Reiner and colleagues [172,173,174]. Studies are currently underway in our lab to examine the potential role of trogocytosis-mediated signaling in the generation of memory CD4^+^ T cells.

## 9. Summary

An apparent paradox exists between the duration of Ag stimulation necessary to fully activate a CD4^+^ T cell and the duration of T cell interactions with an APC. Iezzi et al. showed that full T cell activation requires up to 6 h of sustained Ag stimulation for up to 6 h [175]. However, in an intact lymph node, the duration of initial T–DC interactions is on the order of minutes [176]. In vitro studies have demonstrated that T cells form multiple, short-lived interactions with APC, and that summing of the signals from each of these interactions can lead to full T cell activation [155,156]. These results are consistent with the signaling summation model proposed by Lanzavecchia and colleagues [154]. The Lanzavecchia model requires that the summed signaling events overlap temporally. However, the observed interval between successive T–APC encounters in both in vivo [176] and in vitro studies is on the order of minutes, during which time signaling would likely be terminated. When signaling is terminated prior to full activation, molecules critical for TCR signaling, such as the TCR zeta chain, are partially phosphorylated and the T cells are, therefore, refractory to further stimulation, leading to inactivation of the cells [177]; a phenotype that underlies the TCR antagonism phenomenon [178,179]. Thus, the observed short-duration, repeated T–APC interactions are not strictly in agreement with the Lanzavecchia model. Trogocytosis-mediated signaling, by sustaining intracellular signaling between successive APC interactions, could play an important role in full T cell activation via signal summing.

Trogocytosis-mediated signaling, by altering the activation state, survival, differentiation, and production of effector cytokines, has the potential to significantly impact the physiology and subset differentiation of the trogocytosis-positive cell and, by extension, control the immune response. This paradigm-shifting event may play a significant role in the generation and control of protective immune responses, cancer immunotherapy, and vaccine efficacy. However, we have very limited knowledge of trogocytosis-mediated signaling, and more work is necessary to improve our understanding of this underappreciated phenomenon and the role it plays in modulating the immune response.
